# Participation of adults with disorders/differences of sex development (DSD) in the clinical study dsd-LIFE: design, methodology, recruitment, data quality and study population

**DOI:** 10.1186/s12902-017-0198-y

**Published:** 2017-08-18

**Authors:** Robert Röhle, Katharina Gehrmann, Maria Szarras-Czapnik, Hedi Claahsen-van der Grinten, Catherine Pienkowski, Claire Bouvattier, Peggy Cohen-Kettenis, Anna Nordenström, Ute Thyen, Birgit Köhler, on behalf of the dsd-LIFE group, Birgit Köhler, Peggy Cohen-Kettenis, Annelou de Vries, Wiebke Arlt, Claudia Wiesemann, Jolanta Slowikowska-Hilczer, Aude Brac de la Perriere, Charles Sultan, Francoise Paris, Claire Bouvattier, Ute Thyen, Nicole Reisch, Annette Richter-Unruh, Hedi Claahsen-van der Grinten, Anna Nordenström, Catherine Pienkowski, Maria Szarras-Czapnik

**Affiliations:** 1 Koordinierungszentrum Klinische Studien, (KKS), Charité – Universitätsmedizin Berlin, corporate member of Freie Universität Berlin, Humboldt-Universität zu Berlin, and Berlin Institute of Health, Berlin, Germany; 2 Klinik für Pädiatrie m.S. Pädiatrische Endokrinologie, Charité – Universitätsmedizin Berlin, corporate member of Freie Universität Berlin, Humboldt-Universität zu Berlin, and Berlin Institute of Health, Berlin, Germany; 3 Endocrinologie pediatrique, Centre de reference des maladies rares du developpement sexuel, Hopital Bicêtre, Universite Paris-Sud, Paris, France; 4 Afdeling Kinderendocrinologie, Radboudumc, Nijmegen, The Netherlands; 5 Medische psychologie en medisch maatschappelijk werk, VU Medisch Centrum, Amsterdam, The Netherlands; 6 Department of Women’s and Children’s Health, Karolinska Institutet, Department of Paediatric Endocrinology, Astrid Lindgren Children’s Hospital, Karolinska University Hospital, Stockholm, Sweden; 7 Unite d’Endocrinologie, Genetique et Gynecologie medicale, Hopital des Enfants, Toulouse, France; 8 Clinic of Endocrinology and Diabetology, Children’s Memorial Health Institute, Warszawa, Poland; 9 Klinik für Kinder- und Jugendmedizin, Universität zu Lübeck, Lübeck, Germany

**Keywords:** Disorders of sex development, Differences of sex development, DSD, Sexual differentiation, Interdisciplinary care, European network

## Abstract

**Background:**

dsd-LIFE is a comprehensive cross-sectional clinical outcome study of individuals with disorders/differences of sex development (DSD). This study focuses on various rare genetic conditions characterized by impaired gonadal or adrenal functionality.

**Methods/Design:**

The study aims to assess quality of life (QoL) as a measure of psychosocial adaptation, psychosexual and mental health aspects as major outcomes. Health status and functioning, medical and surgical therapies, participants’ views on health care, psychological and social support, sociodemographic factors and their interrelations will be investigated as factors associated with the outcomes. In addition, ethical considerations in the field of DSD are addressed and previous experiences with health care were gathered. One thousand and forty participants with different DSD conditions were recruited by 14 study centres in 6 European countries (France, Germany, the Netherlands, Poland, Sweden and the United Kingdom) from February 2014 until September 2015. The conditions included were: Turner syndrome (*n* = 301); 45,X0/46,XY conditions (*n* = 45); Klinefelter syndrome (*n* = 218); 47,XYY (*n* = 1); 46,XY gonadal dysgenesis/ovotestes (*n* = 63); complete androgen insensitivity (CAIS) (*n* = 71); partial androgen insensitivity (PAIS) (*n* = 35) and androgen synthesis disorders (*n* = 20); severe hypospadias (*n* = 25); other or non-classified 46,XY DSD (*n* = 8); 46,XX congenital adrenal hyperplasia (CAH) (*n* = 226); 46,XX gonadal dysgenesis/ovotestis (*n* = 21); and 46,XX in males (*n* = 6). For an add-on study, 121 46,XY male-assigned individuals with CAH due to 21-hydroxylase deficiency were recruited. Mean age of participants’ was 32.4 (+/− 13.6 years).

**Discussion:**

Participation was high in conditions not commonly described as DSD, such as Turner and Klinefelter syndromes or CAH. Recruitment of individuals with XY DSD conditions proved to be more difficult. The data collection of PROs resulted in high data quality. Within medical and physical examination data, more missings and/or inaccurate data were found than expected. The European dsd-LIFE study recruited and evaluated the largest cross-sectional sample of individuals with different conditions classified under the term DSD. The data from this large sample will provide a sufficient basis for evidence-based recommendations for improvement of clinical care of individuals affected by a DSD condition.

**Trial registration:**

German Clinical Trials Register DRKS00006072.

## Background

### Rare conditions – Disorders/differences in sex development

“Disorders/differences of sex development (DSD)” is used as an umbrella term for various rare conditions that are characterized by an incongruence of chromosomal, gonadal and genital sex development. The approximate incidence of genital anomalies is estimated to occur 1:4500 live births [1].

The term DSD and a new system for nomenclature were introduced by the Chicago Consensus Group in 2005, replacing previous nomenclature that was perceived negatively by affected individuals. The classification of DSD distinguishes three major groups: (1) DSD with atypical sex chromosome configurations, including Turner syndrome (45,X0 and mosaicisms), Klinefelter syndrome (47,XXY and mosaicisms) and conditions with 45,X/46,XY or 46,XX/46,XY karyotypes; (2) XY DSD, encompassing conditions characterized by 46,XY karyotype and impairment of testicular development, androgen biosynthesis or action, AMH biosynthesis or action, hypospadias, cloacal exstrophy and other syndromic forms; (3) XX DSD, comprising conditions characterized by 46,XX karyotype and androgen excess, such as congenital adrenal hyperplasia, P450 oxidoreductase deficiency, aromatase deficiency or exogenous causes, impairment of ovarian, uterine or vaginal development and other syndromic forms [1]. The multitude of mechanisms related to sex determination and sex differentiation—such as genes involved in gonadal development, androgen receptor function and steroid biosynthesis—makes the diagnoses of the XY DSD group complex and challenging. The heterogeneity of clinical presentations of DSD conditions results in variations of treatment options, including medical, surgical or psychotherapeutic, while their rarity carries the consequence of limited ad hoc knowledge about those various options [2–6]. Clinical studies on DSD are often single-centre experiences from regional samples with a limited number of unambiguous diagnoses of participants, and many of the studies lack appropriate comparison groups. The comparability of these studies is often impeded because different definitions and study methodologies are used [7–19].

The study dsd-LIFE, funded by the European Commission (7th Framework Programme, FP7), has sought to overcome some of the above-mentioned challenges by using a standardized and consistent study methodology in a large number of individuals with DSD conditions in six European countries.

## Methods/Design

### The dsd-LIFE study

**Fig. 1:**
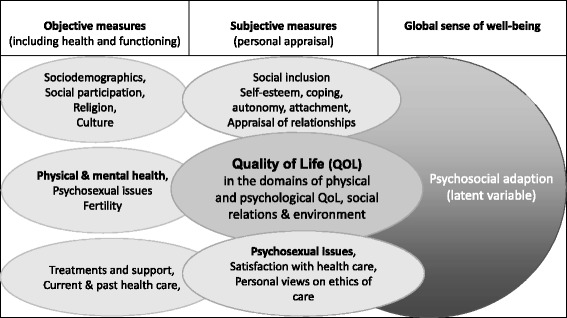
Conceptual model

dsd-LIFE investigates and compares the long-term outcomes of surgical and hormonal therapy and psychological and social support in adequate numbers of adolescents (from the age of 16) and adults with DSD conditions, aiming to provide the basis for improvements in evidence-based recommendations for care. The long-term impact of the study is expected to result in improvement of care and subsequently higher overall quality of life (QoL) and better integration and participation of individuals with DSD in society. To reach these aims, we assessed as main outcomes QoL, psychosexual and mental health aspects. As determinants we collected objective data on sociodemographic factors, social participation, religion, culture, physical and mental health, risk for mental health such as anxiety, depression, autism and ADHD, treatments such as genital surgery and hormone therapy, fertility, psychological and social support, current and past health care, psychological factors such as self-esteem, coping, autonomy, attachment and subjective appraisal of relationships as well as appraisal of relations and personal’ views on ethics of care (Fig. 1).

**Fig. 2:**
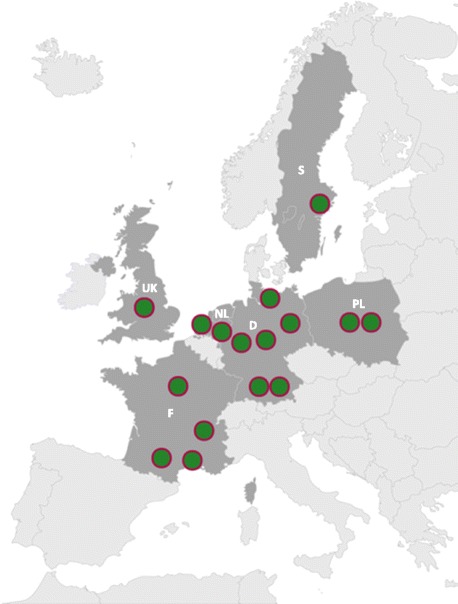
Locations of project partners. The following centres participated in the study: France: Université Claude Bernard Lyon; Le Centre Hospitalier Universitaire Montpellier; Université Paris-Sud, Paris; Le Centre Hospitalier Universitaire de Toulouse. Germany: Charité Universitätsmedizin Berlin; Ludwig-Maximilians-Universität, München; University of Lübeck; Universitätsmedizin Göttingen; Westfälische Wilhelms-Universität Münster. Poland: Medical University of Lodz; Children’s Memorial Health Institute, Warszawa. Sweden: Karolinska Institutet, Stockholm. United Kingdom: University of Birmingham. The Netherlands: VU University Medical Center, Amsterdam; Radboud University Nijmegen Medical Center, Nijmegen

The dsd-LIFE consortium consists of 16 European partners from Germany, France, the Netherlands, Poland, Sweden and the United Kingdom (UK). Fourteen of these partners acted as recruiting sites, including Berlin, München, Lübeck, Münster (D), Paris, Lyon Montpellier and Toulouse (F); Amsterdam and Nijmegen (NL); Lodz and Warszawa (P); Stockholm (S); and Birmingham (UK) (Fig. 2). In addition to the recruiting sites, the consortium included one centre with expertise in medical ethics (Göttingen) and one partner with expertise in European community (EC) project management (München). The Scientific Advisory Board consisted of international experts in the fields of endocrinology, psychology, surgery, and ethics and a representative of a longstanding patient support group for androgen insensitivity syndrome. An important scientific basis for this study was the results of a German network DSD study published in 2009, which was the largest clinical evaluation study of DSD available at the time [20].

In this paper, we outline the theoretical and methodological framework of the study. We compare the study aims related to participation rates and data quality to the actual sample recruited and the information that became available from various sources.

## Conceptual model, study design and methods

### Conceptual model

Historically, clinicians, administrators, researchers, and policy makers have evaluated the effectiveness of treatments and interventions with little input from patients. With the rising participation of patients and clients in shared decision making, evaluation of the impact of care on day-to-day life has become increasingly important. When validated questionnaires are administered directly to participants without involving clinicians, they are called patient-reported outcomes (PROs) and may include reports on objective parameters such as sociodemographic factors, measures of functioning and health status as well as subjective appraisal of one’s QoL and other relevant aspects of well-being. Conceptually, dsd-LIFE developed a framework to determine the major outcome QoL solely from the subjective perspective of the participant and adopted the definition of health proposed by the World Health Organization (WHO), namely, physical, psychological and social well-being (Fig. 1) [21, 22]. For each dimension of QoL, dsd-LIFE sought to collect information from at least one additional perspective. For physical health, dsd-LIFE aimed to collect data from clinicians through medical histories, clinical examinations and data documented in medical records. For psychological health, we probed for indicators of special health care needs, clinical diagnoses and screening instruments of mental health conditions. For social well-being, we collected information on sociodemographics, participation and inclusion [21, 22]. For satisfaction with health care, we measured the subjective rating of the participants of the care they had received, such as satisfaction with medical and psychological care, medical information management, patient-centred care and doctors’ behaviour. To relate outcomes to treatments, we collected medical data of current and past treatments and interventions from medical files as available. The overall aim of the study was to improve health care in the clinical population of individuals with DSD conditions.

### Study design

**Fig. 3:**
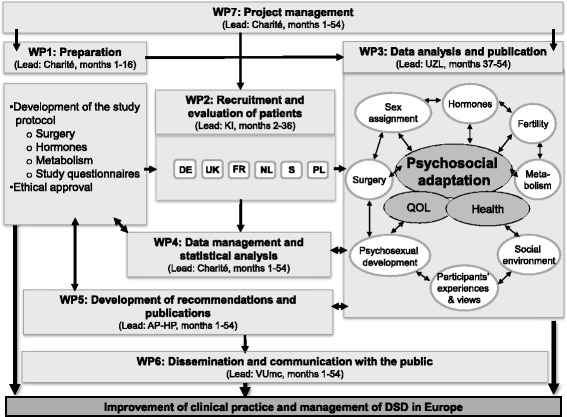
Study design and work packages of dsd-LIFE

dsd-LIFE was designed as a non-interventional, clinical, cross-sectional study. The inclusion criteria covered all conditions described as DSD in the classification system of the Chicago Consensus Conference and an age of 16 years or older. Verification of the correct diagnosis was conducted by examining clinical medical and genetic data; all participants had to be seen at least once at one of the recruiting sites. Health status was assessed by a physical examination, potentially augmented by further examinations. Current and past treatment data were obtained from a medical interview and medical chart review. The wide age range of the subjects allowed assessment and comparison of treatments that were typical in certain decades but changed over time. PRO data came mainly from an online survey, allowing privacy and confidentiality for all participants. The design was chosen to allow the analysis of associations of treatments and interventions experienced in the past with contemporary QoL, physical and mental health, psychosexual functioning and satisfaction with care. In addition, we sought to examine participants’ views and perspectives on the ethics of general health care and controversial treatments (Fig. 3).

We did not recruit a comparison or control group, but results of major outcomes will be compared to reference data. Moreover, we planned to use group comparisons of the diagnoses within the sample because some issues such as e.g. fertility or psychosexual problems are, to varying degrees, issues for all people affected by DSD.

### Instruments

The use of available, standardized instruments with reference data from the general population was preferred whenever possible, thus allowing comparisons to the general population, specifically in the areas of sociodemographics, QoL and screening measures of physical and mental health, psychosexual outcome, psychological factors and overall satisfaction with health care. For specific aspects of DSD, we included “self-constructed” items. The iterative review of the suggested instruments and measurements was performed by experienced patient support group representatives and by the project’s Advisory Board.

For application of the final questionnaire in all study-relevant languages (Dutch, English, French, German, Polish, and Swedish), professional translation of the instruments used was required. To some extent, the standardized instruments chosen were available in the languages needed (Table 1). For those instruments and (self-constructed) items that required translation, we followed the linguistic validation process of the international translation guidelines [23, 24].

**Table 1:** Instruments used in dsd-LIFE

Construct	Instrument	Domain (number of items)	Response scales	Translation required^a^	Reference
Sociodemographics					
Sociodemographic information	European Social Survey (ESS)	Area of residence (1: ESS); Education (2: ESS, 1: SC^b^); Household and family (1: ESS, 8: SC); Occupation (3: ESS); Nationality (3: ESS, 2: SC); Religious denomination (3: ESS); Participation/“you and other people” (5: ESS, 3: SC)	4- to 7-pt. Likert Scales Dichotomous (yes/no) Free text	None	[42]
Quality of life					
Quality of life	WHO-QOL-Bref	Physical health (7); Psychological QOL (6); Social relationships (3); Environment (8) Global (2)	5-pt. Likert Scale	None	[8, 26, 50]
General health					
General health Condition related health issues	EHIS (European Health Interview survey), self-constructed	1 EHIS; 2 SC	3- to 5-pt. Likert Scale Dichotomous (yes/no) Free text	None	
Mental health and psychological factors					
Mental Health					
	EHIS, self-constructed	10 EHIS, 36 SC	4-pt. Likert Scale, dichotomous		
Screening for anxiety and depression	Hospital anxiety and depression scale (HADS)	Anxiety (7); Depression (7)	4-pt. Likert Scale	None	[28]
Screening for ADHD	Adult ADHD Self-Report Scale (ASRS-v1.1)	ADHD (6)	5-pt. Likert Scale	Polish	[51–53]
Screening for autism	AQ-10	Autism (10)	5-pt. Likert Scale	Dutch, French, German, Polish	[54]
Psychological factors					
Self-esteem	Rosenberg self-esteem	Self-esteem (10)	4-pt. Likert Scale	None	[55, 56]
Coping	DSD-questionnaire for adolescents	Shame and stigmatization (5); Openness (4); SC (4)	4-pt. Likert Scale	Dutch, French, German, Polish, Swedish	[57]
Autonomy	ESS	Autonomy (4)	5-pt. Likert Scale	None	[42]
Attachment style	ECR-RS	Avoidance (6), Anxiety (3)	7-pt. Likert Scale	Dutch, French, German, Polish, Swedish	[58, 59]
Psychosexual issues					
Body perception and feelings	Body Image Scale (BIS)	(36)	5-pt. Likert Scale	French, Polish, Swedish	[29, 30]
Sexuality	Self-constructed	Satisfaction with frequency of sexual activity (1), with sex life in general (1), and with sexual functioning (1); Sexual frequency (1); Sexual problems and suffering (1); Sexual orientation (1)	5-pt. Likert Scale Dichotomous (yes/no) Free text	Dutch, French, German, Polish, Swedish	
Stages of intimate relationships	Self-constructed	16 items	Dichotomous (yes/no) Number	Dutch, French, German, Polish, Swedish	
Gender dysphoria in adolescents and adults	Utrecht Gender Dysphoria Scale (UGDS)^c^	Screening (1), Female version (10), Male version (10)	5-pt. Likert Scale	French, Polish	[60, 61]
Feelings of masculinity and femininity	Self-constructed	2 items	10-pt. Likert Scale	Dutch, French, German, Polish, Swedish	
Current care and support					
Utilization of and satisfaction with care	Adapted CHC-SUN Self-Report Version (Modules 1 and 2)	Utilization (4), SC (10) Satisfaction with care: diagnosis/information (2 + 2 SC), patient-centred care (3), doctor’s behaviour (3)	5-pt. Likert Scale	Dutch, French, German, Polish, Swedish	[35, 36]
Satisfaction with care	CSQ-4	(4)	4-pt. Likert Scale	None	[62, 63]
Satisfaction with DSD specific care	Self-constructed	(3)	5-pt. Likert Scale Dichotomous (yes/no) Free text	Dutch, French, German, Polish, Swedish	
Past care and support					
Information management	Self-constructed	Diagnosis (1); Disclosure and understandability (14)	4- to 7-pt. Likert scales Dichotomous (yes/no) Free text	Translation needed for all countries	
Treatment	Self-constructed	Treatment location (1), Satisfaction with treatment (1), Psychological care (1), Satisfaction with psychological care and General support (3)	4- to 7-pt. Likert scales Dichotomous (yes/no) Free text	Translation needed for all countries	
Hormone treatment	Self-constructed	Screening question on hormone treatment received (1): Oestrogen, Oestrogen-Progestin combination, Testosterone, Dihydrotestosterone (DHT), Cortisone, Fludrocortisone, Growth hormone, GnRH-Agonist, Treatment for tall stature, Anti-androgens, Aromatase inhibitors	Dichotomous (yes/ no) Free text	Translation needed for all countries	
Satisfaction with hormone treatment (per hormone and with respect to side effects)	Self-constructed	Timing of treatment (6), Satisfaction with treatment in terms of associated side effects (4)	3- and 5-pt. Likert scale Free text	Translation needed for all countries	
Performed surgery and/or medical procedures	Self-constructed	Screening question on performed procedure, including age and patient consent: Vaginoplasty, Clitorectomy/clitoral reduction, Vaginal dilations, Hypospadias repair, Relocation of testes, Removal of the gonads, Removal of the uterus, Breast removal, Breast enlargement,	Dichotomous (yes/no)	Translation needed for all countries	
Satisfaction with medical treatment	Self-constructed	Number of treatments (1), Surgery-related complications (1), Satisfaction after surgery/functionality (2), Impact on life (3)	Tick box 5- and 6-pt. Likert Scale Free text	Translation needed for all countries	
Fertility	Self-constructed	Communication and satisfaction with communication (5), Fertility (4)	Dichotomous (yes/no) 5-pt. Likert Scale Free text	Translation needed for all countries	
Patients’ views and opinions on DSD					
Ethics	Self-constructed	Care and support (7), Patient’s rights (12), Society (8)	Different Scales	Dutch, French, German, Polish, Swedish	
Terminology	Self.constructed	Satisfaction with medical terminology (3)	5- and 10-pt. Likert Scale Choice of 32 condition-specific terms	Dutch, French, German, Polish, Swedish	

^a^Performed after international translation standards

^b^SC = self-constructed

^c^To use the Utrecht Gender Dysphoria Scale in dsd-LIFE, two items were deleted in both the male and female versions of the questionnaire (UGDS-M original question: No. 9: I dislike urinating in the standing position, No. 10: I am dissatisfied with my beard growth because it makes me look like a man; UGDS-F original question: No. 8: I enjoy seeing my naked body in the mirror, No. 10: I hate menstruating because it makes me feel like a woman)

#### Medical interview, retrospective chart review and medical examinations (part 1)

Generally, the data came from a personal medical review on contemporary signs and symptoms, past medical history and a physical examination with a physician; the data were input by a study nurse or physician (case report form; CRF):

1. The medical interview (239 items) collected standardized information on the diagnosis, karyotype and comorbidities of the participants and their families, physical activity, smoking behaviour, current therapy, application, therapy monitoring, previous hormone therapies and psychological counselling and menstruation.

**A retrospective chart review** of past diagnosis and medical treatment was performed to obtain information from medical records, including birth history and genital phenotype at diagnosis, date of diagnosis, and hormones and hormone therapy at diagnosis (dosage, application) **(79 items)**. Previous surgical procedures and complications were also retrieved from medical records **(29 items)**. Further retrospective data related to hormone treatment were gathered from medical records at the time of puberty (Tanner stage II) (6 items) and completion of puberty (> = 16 years) **(45 items)**. For individuals with congenital adrenal hyperplasia (CAH), additional data on hormone therapy were taken from the records at the ages of 9 months and 6 years to evaluate steroid hormone levels and treatment outcomes **(30 items each)**. Moreover, descriptions of ambiguity of genitalia (44 items) were gathered from the records, if applicable.

2. The **general physical exam** was used to obtain a general impression of the patient; anthropometric measures of height, weight, body mass index (BMI), and hip and waist circumference; and blood pressure (mean of 3 measurements). The exam also revealed the presence of acanthosis; stretch marks; acne; hirsutism; decrease of body hair; bruised skin; and pubic hair. Breast development was assessed according to the Tanner and Ferriman Galway score **(13 items)**.
3. An optional **gynaecological/urological** examination, including a gynaecological history if applicable, general genital development and surgical results, was performed for participants who had undergone genital constructive surgery **(11 gynaecology/9 urology items)**.
4. The **laboratory investigations featured blood tests** that used a fasting blood sample to measure metabolic parameters such as lipids, glucose, insulin, R-Homa, liver enzymes, uric acid, whole blood count, and renal parameters for all diagnostic groups; hormones in all females: gonadotropins, oestrogen, androgens, and thyroid function; hormones in all males: gonadotropins, oestrogen, androgens, anti-muellerian hormone (AMH), inhibin B and thyroid function; and hormones in all participants with CAH, including 17-hydroxyprogesterone, androstenedione, and renin. In participants with gonads in place, AMH, inhibin B and gonadal tumour markers were measured **(38 items)**.
5. **The optional technical assessments** included dual-energy X-ray absorptiometry (Dexa-scans) measuring bone mineral density and body composition **(15 items);** bioelectrical impedance analysis (BIA) evaluating body composition **(12 items)**; ultrasound of the carotic artery measuring intima media thickness (IMT) **(9 items)**; ultrasound of the uterus and ovaries estimating uterine size to evaluate the development and oestrogen effects and the ovarian structure and possible abnormalities; testicular ultrasound measuring the testicular size and structure and possible abnormalities **(57 items)**; and a spermiogramme evaluating sperm quality (count, motility, morphology, vitality, and volume) **(6 items)**.

#### Patient-related outcomes (PROs, part 2)

This part of the study included standardized instruments and self-constructed questionnaires (Table 1).

1. **Sociodemographic data** such as age, area of dwelling, education, household and family, occupation, nationality, religious denomination and social participation were gathered according to the European Social Survey (ESS; source questionnaire amendment 01, 2012/2013). All questions were provided in interview format to maximize the accuracy and objectivity of the information. For dsd-LIFE, we transformed these questions into self-reporting questionnaires, retaining the response format. Validated translations and reference data are available for all countries of the dsd-LIFE consortium (http://www.europeansocialsurvey.org).
2. For the assessment of **QoL**, we used the WHOQOL-BREF, which evaluates physical and **psychological QoL, social relationships, environment and global QoL**; reference data on both the general population and clinical samples for comparison are available [25, 26].
3. To assess **physical health** we used questions from the European Health Interview Survey (EHIS) and self-constructed condition specific questions.
4. To evaluate **mental health** we used questions from the EHIS and self-constructed questions. We used the Hospital Anxiety and Depression Scale (HADS) to screen for depression and anxiety. We used the Autism Spectrum Quotient (AQ)-10 test and the Adult ADHD Self-Report to screen for signs and symptoms of for autism and ADHD as they have been described as common comorbidities in the DSD population in previous studies [27, 28].
5. To asses **psychological factors** the following questionnaires were chosen: the Rosenberg Self-esteem Scale (RSES) for **self-esteem,** a self-constructed questionnaire **for shame, stigma and coping**, questions form the ESS for **autonomy**, the Relationship Structures (ECR-RS) Questionnaire for **attachment**.
6. **Psychosexual issues** were evaluated by standardized and condition-specific self-contructed questionnaire. The Body Image Scale (BIS) was chosen to investigate **body satisfaction**, an issue of high relevance in the condition groups studied [29, 30].
  We opted to evaluate **gender dysphoria** with the well-established and relatively short Utrecht Gender Dysphoria Scale (UGDS). This instrument has been developed for the assessment of people without DSD who have gender identity disorder but has been used in previous studies in DSD [31]. In addition, **feelings of male- and femaleness** were evaluated by two self-constructed questions. We chose not to evaluate gender role behaviour, which shows a wide variation in the normal population and does not have clinical relevance in itself.
  The questionnaire on **sexuality** was developed for the German network study evaluating developmental milestones sexual problems, feelings and satisfaction with sexual life. The items had performed positively in that study, and the scale was thus selected and translated into the various languages for dsd-LIFE [10, 32]. **Sexual orientation** was measured with the Kinsey scale, and the wording was changed to make the questions less dichotomous in terms of gender [33, 34].
7. For evaluation of **satisfaction with health care,** the Client Satisfaction Questionnaire (CSQ4) and an adapted version of the Child Health Care – Satisfaction, Utilization, and Needs Questionnaire (CHC-SUN) was chosen. The CHC-SUN was originally developed as a proxy-report version for parents of children with chronic conditions and later adapted and tested as a self-report version for young adults with chronic diseases. For this study, an extended short form of the CHC-SUN was applied, and several self-constructed items on satisfaction with care and needs were added [35, 36]. For assessment of **current and past health care**, satisfaction with information management, satisfaction with hormone therapies, genital surgery, current and previous treatment/psychological/social support and fertilty self-constructed questions were used.
8. Participants’ perspectives on **ethical issues** were assessed by self-constructed questionnaires. The items on ethical issues had been developed by the ethical research group in Göttingen based on qualitative work with focus groups and individual interviews. The interview questions were closely related to the recommendations for care of individuals with DSD, which were developed by the German Ethical Council in 2012 [37, 38]

**Fig. 4:**
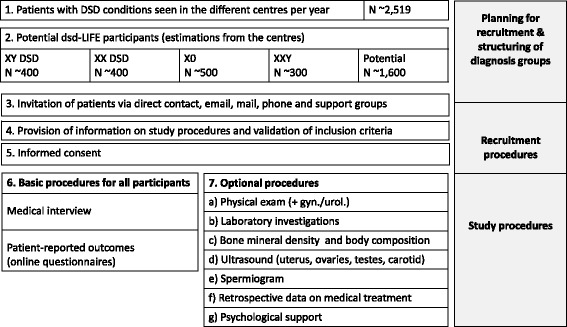
Study procedure

Obligatory requirements for participation in the study were participation in the medical clinical interview and completion of the PRO questionnaire. Medical examinations were considered an important part of the study but were not obligatory. Participants not interested in medical examinations were included into the study if they met the above requirement, if they had visited the centre in person at least once, and if they had documented previous clinical consultations and diagnosis (Fig. 4).

### Inclusion criteria

The study included individuals with a confirmed diagnosis of DSD with variants in sex chromosomes, including Turner syndrome, Klinefelter syndrome and mixed gonadal dysgenesis (45,X/46,XY); XY conditions, including testicular dysgenesis, impairment of testosterone synthesis or action and hypospadias; XX conditions, including ovarian dysgenesis, congenital adrenal hyperplasia (CAH), and XX males; and ovotesticular DSD. XY male participants with CAH (21-OHD and 11ß-HSD deficiency) were included, although, according to the definition, they do not belong to the DSD classification. This group was an add-on study population because it was hypothesized that in many ways these subjects might face similar problems as persons within the DSD classification, such as e.g. sex hormone imbalances and fertility problems. Participants had to be at least 16 years old with a medically confirmed clinical and/or genetic diagnosis (Fig. 4). The age limit of 16 years was chosen because pubertal development is essential for the evaluation of psychosexual development. Moreover, the initial effects of treatment in childhood and adolescence on the metabolic system can be evaluated at the beginning of adulthood. In addition, the perspectives of adolescents regarding their DSD condition and treatment are of particular interest.

### Exclusion criteria

Individuals not fulfilling the diagnostic criteria or incapable of giving consent or answering the questions on their own were excluded. Subjects with Mayer-Rokitansky-Küster-Hauser syndrome (uterus agenesis) and other complex non-endocrine urogenital malformations were also excluded as most centers had no access to patients with these diagnoses.

### Ethics

Ethical approval was first sought from the medical ethics committee at the Charité Universitätsmedizin Berlin. Ethical approvals were subsequently sought from the other study centres. Data protection and valid procedures to secure the anonymity of the participants were crucial requirements of the study and for the ethical approval. Dsd-LIFE participants were informed thoroughly about the study content, aims and potential risks and subsequently gave written informed consent. The informed consent was written in lay language to ensure the understandability of the aims, procedures and potential risks of participation. All participants had to consent to the release of their medical data retrieved from medical charts, including genetic test results. If the participant was underage, both the potential participant and the parents of the participant had to give written informed consent.

The opportunity for withdrawal from the study was possible at any stage of the study. Travel costs of the participants were covered upon request. According to the World Medical Association’s Helsinki Declaration and the Council of European Convention on Human Rights and Biomedicine [39], dsd-LIFE posed minimal risk for the participants (e.g., medical exams, blood sampling) [40, 41].

### Recruitment

The 14 recruiting centres approached former and current patients with a diagnosis meeting the inclusion criteria for dsd-LIFE and promoted participation in the study in various additional ways. The means of contacting potential study participants included regular mail, e-mail, phone, direct contact of the physician (at consultation hours at the recruitment site or at consultation hours of regional endocrinologists linked to the recruitment site), patient support groups and information from the dsd-LIFE website (www.dsd-life.eu). A standardized record was kept by each centre to document the recruitment procedure and the informed consent process. In this way, the process of individual approach and information about acceptance or denial of participation and inclusion were retained. Participant recruitment started on February 1, 2014 and ended on September 30, 2015. To obtain adequate numbers in the XY DSD group, recruitment for this group was prolonged from 16 to 20 months.

### Study logistics

Each recruiting centre established a team to contact, include and monitor participants throughout the study. Study teams could consist of endocrinologists, gynaecologists, urologists, surgeons, psychologists, and study nurses: usually, the endocrinologist informed the potential participant, assured or verified consent and subsequently performed the medical examination. Gynaecologists, urologists or surgeons accomplished the gynaecological or urological examinations as required and if the participant had consented to these examinations. All clinicians provided counselling for treatment options if needed or arranged for appropriate referrals. The study nurse or physician entered the data of the medical CRFs.

The PRO was explained by the study nurse. To ensure confidentiality and unbiased responses, participants were asked to respond to the online version of the PRO, which was accessible only with a secure password in the recruitment centres. A physician, and when possible a psychologist, was available for questions and to support the participant at any time during the study. In case the participant was not able or willing to complete the PRO at the recruitment centre, online access was provided for answering the questions at home. If needed, a paper-and-pencil version was provided for the participant, and data entry was accomplished by the study nurse after the patient had completed the questions.

### Training and quality management

All of the study procedures, beginning with enrolment, were set up according to standard operation procedures (SOP). Each recruitment team received digital and printed booklets with the SOPs and was trained before recruitment started. The training occurred either face-to-face in a local study centre or via Skype conferences. The training and the SOPs addressed all relevant study procedures, including the inclusion or exclusion criteria; organizational aspects (patient information, informed consent, pseudonymization of data, maintenance of study records, patient time plan, travel reimbursement, contact details for participants); standardized performance of medical exams; blood sampling and biobanking; registration of core data on the diagnosis in the international DSD patient registry, if the participant consented (I-DSD, https://www.i-dsd.org/); administration of PROs and CRFs; data entry; and handling of information associated with exclusion from the study or the decision not to approach a potential participant. Such information was held at the discretion of the recruiting centre. For ethical reasons and data protection, we were not allowed to enter data on diagnosis, age or gender or other information available from the charts in the dsd-LIFE database if the patient had not consented to participate in the study. Therefore, data on non-responders are not available in a systematic manner. To ensure ongoing recruitment and timely study performance and to ensure data quality at data entry, the study management (Work Package 2, Fig. 4) liaised regularly with each recruitment centre.

### Data management, safety and quality

The data collected during the study were entered into a central database using electronic data capture. The data were entered remotely at the sites using electronic CRFs that also allowed definition of specific roles (clinical investigator, study assistant, etc.) to control access rights depending on the function within the study. To ensure data safety and data privacy, personal data were pseudonymized. Only the study data of the participants were stored in the study database. Personal data of the individuals were not saved in the database at any time. The study data could be linked to the personal data only by using the pseudonyms, which were created automatically by the system. A pseudonymization list was safely stored in the local study centres and could be accessed only by the local principal investigator. Data checks were performed at regular time points (every 3–6 months) during recruitment. At the end of data entry, the filling status of medical data and the participant questionnaires were checked. Data plausibility, data consistency, and the possibility of missing data were verified. In addition, checks were performed to identify entire empty datasets, non-empty datasets with missing data and other implausibilities and inconsistencies. Queries regarding possible data entry errors or missing values were sent to the clinical sites for clarification or completion, respectively.

The Coordinating Centre for Clinical Studies at the Charité (Koordinierungszentrum für klinische Studien, KKS Charité) performed data management and quality assurance.

All diagnoses were verified at the end of recruitment by the leading study centre at Charité Universitätsmedizin Berlin. The complex diagnoses included in the XY DSD and the CAH groups (B.K., M.S.-C., H. C.-G., A.N.) were double checked by two endocrinologists independently. Implausibilities were discussed with the local study centre.

### Statistical analysis of the dsd-LIFE cohort

To describe the dsd-LIFE study group in detail, we first compared the planned, recruited and participating numbers of participants, in total and per diagnostic group (Fig. 4). The recruitment of individuals was described using absolute and relative frequencies of contacted persons and participants in total and per country. Information on “recruitment ways” (the ways potential participants were contacted) were collected from the participating sites; however, due to missing data, it was not possible to provide a comprehensive quantitative analysis. Relevant participant characteristics of the recruited study population were analysed descriptively in total and per country using means and standard deviations for continuous parameters and absolute and relative frequencies for categorical parameters. Relevant social characteristics of the dsd-LIFE cohort were compared with the results from the population-based European Social Survey for the countries involved in dsd-LIFE comparison samples [42].

To assess data quality, the number of non-responses to entire questionnaires and sub-domains was examined by calculating relative frequencies in the total cohort and in the diagnostic sub-groups. Participation in the medical examinations and the availability of retrospective data were assessed using relative frequencies per diagnosis group, per country and per gender where appropriate. All computations were performed using R (Version 3.2.2) and statistical analysis system (SAS Version 9.4).

## Results

### Description of study population

#### Recruitment

At the beginning of the project, the recruitment sites reported a total number of 2519 persons with the different DSD diagnoses currently treated in the centres or who had been treated in the past. Based on that number and considering loss to follow-up, exclusion criteria and those who declined to participate, the centres estimated 1697 potential participants in 2012 (Fig. 3). However, in the project recruitment phase (2/2014–9/2015), the centres were able to identify and to invite 3217 potential participants from their records, and 1161 agreed to participate in the study (36.1%). Most of the 1161 dsd-LIFE participants were recruited in France (*n* = 311, 26.8%), followed by Germany (*n* = 291, 25.1%), the Netherlands (*n* = 265, 22.8%), Sweden (*n* = 131, 11.3%), Poland (*n* = 108, 9.3%), and the United Kingdom (*n* = 55, 4.7%) (Table 2).

**Table 2:** Participation rate in dsd-LIFE

Study sites	Contacted *n* = 3217	Participation *n* = 1161 (36.1%)		Non-participation *n* = 2057 (63.9%)	
Germany	927	291	(31.4%)	636	(68.6%)
France	676	311	(46.0%)	365	(54.0%)
The Netherlands	882	265	(30.0%)	617	(70.0%)
Poland	331	108	(32.6%)	223	(67.4%)
Sweden	241	131	(54.4%)	110	(45.6%)
The United Kingdom	160	55	(34.3%)	105	(65.7%)

#### Diagnosis

Among the 1161 participants, 1040 had a DSD condition according to the Chicago classification (Table 3), and 121 were males with 21-hydroxylase deficiency or 11-β-hydroxylase deficiency. Five participants had to be excluded during the verification process of diagnoses as non-cases. Overall, the project achieved a participation rate of 36.1%, ranging from 54.4% in Sweden to 30.0% in the Netherlands. An overview of participation by country is given in Table 2. From 3217 approached persons, the recruitment means and respective participation rate could be tracked for 1717 individuals. The numbers of contacted persons/numbers of participants/participation rate were as follows: by mail: 1222/268/22%, e-mail: 40/25/63%; telephone: 91/40/44%; personal contact in the clinic: 282/215/76%; and support groups: 82/20/24%. Of these methods, response rates (76%) were highest among persons who were contacted directly by their physician. The recruitment of individuals with a diagnosis of sex chromosome DSD was achieved as intended, whereas recruitment of individuals with 46,XY DSD diagnosis and 46,XX gonadal dysgenesis diagnosis resulted in approximately half the initially planned numbers. The diagnoses and gender and of the cohort are described in detail in Table 3. Proportions of diagnoses and gender are shown in Table 6. The participants with Turner syndrome were classified according the Mortensen classification [43]. In the groups of participants with Klinefelter and Turner syndrome, the clinical diagnosis was confirmed genetically in almost all cases (>96%). In the heterogeneous groups of diagnoses summarized under XY DSD and XX CAH, the proportions of genetically confirmed diagnoses were 47.5% and 69%, respectively. However, the precise mutation responsible for the genetic conditions was not documented in the patient files in some centres due to insufficient or lack of transmission from the genetic laboratory. (In some countries, sharing genetic information with clinicians requires written informed consent by the patient.) In the group of XX gonadal dysgenesis, only clinical diagnoses were documented. Detailed information on the proportions of genetic and clinical diagnoses is shown in Table 4. In participants with CAH due to 21-hydroxylase deficiency (21-OHD), the severity of the enzyme defect was classified according to the clinical phenotype. In addition, this group was classified according to genotype because a valid genotype-phenotype correlation could be demonstrated. For 21-OHD, genotype-phenotype classification was established ranging from 0 for salt-wasting for the severest forms to C for non-classical, which is the least severe form [44, 45]. This classification according to the genotype of participants with 21-OHD deficiency is presented in Table 5.

**Table 3:** Classification of participants according to the Chicago classification

Diagnosis group	n	Classification	Gender			Sum
			Female	Male	Other	
Sex chromosome DSD	301	Turner syndrome				
		Monosomy: 45,X	150	0	0	150
		Mosaics: 45,X/46,XX	31	0	0	31
		Isochromosomes: 45,X/46,X,i(Xq) | 46,X,i(Xq) | 45,X/46,X,i(Xq)/47,X,i(Xq)	59	0	0	59
		Deletions: 45,X/46,X,del(X) | 46,X,del(X)	19	0	0	19
		Polyploidy: 45,X/46,XX/47,XXX | 45,X/47,XXX | 45,X/46,XX/47,XXX/48,XXXX	16	0	0	16
		Ring material: 45,X/46,X,r(X)	12	0	0	12
		Not classified	4	0	0	4
		Unknown	10	0	0	10
	45	45,X/46,XY conditions	31	14	0	45
	218	Klinefelter syndrome				
		47,XXY	1	199	4	204
		47,XXY/46,XY	0	5	1	6
		47,XXY/46,XX	0	3	0	3
		Other	0	3	0	3
		Unknown	0	2	0	2
	1	47,XYY^a^	0	1	0	1
XY DSD	222	XY, Complete GD	20	0	1	21
		XY, Partial GD	12	25	0	37
		XY, Ovotesticular DSD	3	2	0	5
		CAIS	69	0	2	71
		PAIS	17	18	0	35
		3ß-HSD deficiency	1	1	0	2
		17β-HSD III deficiency	9	0	2	11
		5α-RD II deficiency	2	1	1	4
		17α-hydroxylase/17,20-lyase deficiency	1	0	0	1
		Unknown steroid synthesis defect	1	1	0	2
		Hypospadias	0	24	1	25
		XY DSD not classified	7	1	0	8
XX DSD	21	XX gonadal dysgenesis	20	0	0	20
		XX ovotesticular DSD	1	0	0	1
	226	CAH (21-hydroxylase deficiency)				
		Salt-wasting	109	2	0	111
		Simple virilizing	65	1	0	66
		Non-classical	33	1	0	34
		not to classify	3	0	0	3
		Other CAH				
		STAR deficiency	1	0	0	1
		3β-HSD deficiency	2	0	0	2
		11β-hydroxylase deficiency	5	1	0	6
		POR deficiency	2	0	0	2
		Unknown	1	0	0	1
	6	46,XX testicular and unknown DSD	0	6	0	6
		Sum	717	311	12	1040

*GD* gonadal dysgenesis, *PAIS* partial androgen insensitivity, *CAIS* complete androgen insensitivity, 3ß-HSD 3β-hydroxysteroid dehydrogenase, *17β-HSD* *III* 17β hydroxysteroid dehydrogenase III, *5α-RD* *II* 5α-reductase II, *STAR* steroidogenic acute regulatory protein, *POR* cytochrome P450 oxidoreductase. ^a^The patient with 47,XYY, although not belonging to the Chicago classification, is included, as he displays gonadal dysgenesis as Klinefelter syndrome

**Table 4:** Number of genetically and clinically confirmed diagnoses of XY DSD and CAH

Classification of conditions	Conditions	Number of participants	Genetically verified diagnosis		Clinical diagnosis^a^	
Sex Chromosome DSD	Turner syndrome	301	291	(96.7%)	10	(3.3%)
	Klinefelter syndrome	218	216	(99.1%)	2	(0.9%)
	45,X/46,XY	45	45	(100%)	-	(0%)
	47,XYY	1	1	(100%)	-	(0%)
XY DSD	Complete XY GD	21	11	(52.4%)	10	(47.6%)
	Partial XY GD	37	7	(18.9%)	30	(81.1%)
	XY ovotesticular DSD	5	2	(40.0%)	3	(60.0%)
	CAIS	71	50	(70.4%)	21	(29.6%)
	PAIS	35	18	(51.4%)	17	(46.6%)
	3β-HSD	2	2	(100%)	-	(0%)
	17β-HSD III	11	11	(100%)	-	(0%)
	5α-RD II	4	4	(100%)	-	(0%)
	17α-hydroxylase/17,20-lyase	1	-	(0%)	1	(100%)
	Other androgen synthesis defects	2	-	(0%)	2	(100%)
	XY DSD not classified	8	-	(0%)	8	(100%)
	Severe hypospadias	25	-	(0%)	25	(100%)
XX DSD	XX GD	20	-	(0%)	20	(100%)
	XX ovotesticular DSD	1	-	(0%)	1	(100%)
	CAH	226	156	(69.0%)	70	(31.0%)
	XX males	6	3	(50.0%)	3	(50.0%)

In the add-on group of 121 XY CAH, diagnoses were genetically verified in 81.8%; ^a^Diagnosis based on clinical and biochemical findings without positive genetic test results

**Table 5:** Classification of individuals with 21-OHD (CYP21A2) and other CAH according to genotype

Total	0	A	B	C	No mutation or not classified	Other CAH	Sum
All	55	81	75	30	92	14	347
46,XX	31	44	42	27	70^a^	12^b^	226
46,XY	24	37	33	3	22^c^	2^d^	121

This classification includes severity of 21-OHD according to genotype. 21-OHD shows a spectrum ranging from 0 = salt-wasting to C = non-classical when performing genotype-phenotype correlations

^a^These include no mutation (*n* = 68) and “not classified” (*n* = 2)

^b^These include STAR defect (*n* = 1), POR deficiency (POR) (*n* = 2), 3ß-HSD deficiency (HSD3B2) (*n* = 2), 11ß-OHD deficiency (CYP11B1) (*n* = 6) and unknown other (*n* = 1)

^c^These include only no mutation (*n* = 22)

^d^These include 11ß-OHD deficiency (CYP11B1) (*n* = 2)

#### Sociodemographics

The sociodemographic characteristics of the 1040 participants with a DSD diagnosis in the dsd-LIFE cohort are shown in detail in Table 6. Data of the 121 males with CAH are not shown. Missing values for sample characteristics were generally below 10%, with no missing data regarding age or gender and moderate missing frequencies (>10%) for working hours per week (290/1040, 27.9%) and feelings about household income (164/1040, 15.8%).

**Table 6:** Characteristics of the study sample (overall and in all dsd-LIFE countries)

Variable	Levels	Overall	DE		FR		NL		PL		SE		UK	
Total sample		1040	244	ESS	274	ESS	250	ESS	107	ESS	122	ESS	43	ESS
Diagnosis group	Turner	301 (28.9)	43 (17.6)		116 (42.3)		82 (32.8)		3 (2.8)		46 (37.7)		11 (25.6)	
	Klinefelter	218 (21.0)	38 (15.6)		26 (9.5)		88 (35.2)		23 (21.5)		35 (28.7)		8 (18.6)	
	47,XYY	1 (0.1)	0		0		1 (0.4)		0		0		0	
	XY DSD	222 (21.3)	57 (23.4)		42 (15.3)		46 (18.4)		54 (50.5)		18 (14.8)		5 (11.6)	
	XX gonadal dysgenesis	21 (2.0)	4 (1.6)		10 (3.6)		1 (0.4)		2 (1.9)		4 (3.3)		0	
	CAH	226 (21.7)	91 (37.3)		62 (22.6)		27 (10.8)		15 (14.0)		12 (9.8)		19 (44.2)	
	46,XX male	6 (0.6)	3 (1.2)		0		1 (0.4)		0		2 (1.6)		0	
	45,X/46,XY + other Y	45 (4.3)	8 (3.3)		18 (6.6)		4 (1.6)		10 (9.3)		5 (4.1)		0	
Age mean (sd)		32.36 (13.57)	32.36 (13.46)	48.70 (18.57)	25.59 (8.63)	51.82 (18.48)	39.37 (14.70)	51.17 (17.99)	25.18 (10.13)	46.10 (18.85)	38.62 (13.21)	47.83 (19.01)	34.79 (11.79)	51.83 (19.12)
Age groups	<= 19 years	193 (18.6)	41 (16.8)		78 (28.5)		26 (10.4)		42 (39.3)		5 (4.1)		1 (2.3)	
	20–24 years	185 (17.8)	47 (19.3)		68 (24.8)		28 (11.2)		22 (20.6)		14 (11.5)		6 (14.0)	
	25–44 years	458 (44.0)	110 (45.1)		118(43.1)		100 (40.0)		37 (34.6)		65 (53.3)		28 (65.1)	
	45–64 years	178 (17.1)	40 (16.4)		10 (3.6)		84 (33.6)		5 (4.7)		32 (26.2)		7 (16.3)	
	> = 65 years	26 (2.5)	6 (2.5)		0		12 (4.8)		1 (0.9)		6 (4.9)		1 (2.3)	
Gender	Female	717 (68.9)	182 (74.6)	49.6	231 (84.3)	55.2	153 (61.2)	53.6	45 (42.1)	52.1	73 (59.8)	48.7	33 (76.7)	57.4
	Male	311 (29.9)	56 (23.0)	50.4	43 (15.7)	44.8	91 (36.4)	46.4	62 (57.9)	47.9	49 (40.2)	51.3	10 (23.3)	42.6
	Open	2 (0.2)	1 (0.4)		0		1 (0.4)		0		0		0	
	Third	7 (0.7)	5(2.0)		0		2		0		0		0	
	Other (“human being”, “varies”, “other”)	3 (0.3)	0		0		3 (1.2)		0		0		0	
Citizenship *n* = 991 (95.3%) NA = 49 (4.7%)	G, F, NL, P, S, UK	970 (97.9)	226 (9.6)	96.3	250 (98.8)	96.1	230 (99.6)	98.7	106 (99.1)	100.0	119 (100.0)	97.8	39 (92.9)	97.2
	Other EU country	8 (0.8)	7 (2.9)	1.3	0	1.5	0	0.6	0	0.0	0	0.8	1 (2.4)	1.0
	Non-EU country	13 (1.3)	6 (2.5)	2.3	3 (1.2)	2.4	1 (0.4)	0.7	1 (0.9)	0.0	0	1.4	2 (4.8)	1.8
Language spoken at home *n* = 988 (95.0%) NA = 52 (5.0%)	Same as in home country	927 (93.6)	217 (91.2)		236 (94.0)		220 (95.2)		106 (99.1)		111 (93.3)		37 (88.1)	
	Not the same	61 (6.2)	21 (8.8)		15 (6.0)		11 (4.8)		1 (0.9%)		8 (6.7)		5 (11.9)	
	dsd-LIFE country	17 (27.9)	6 (28.6)		2 (13.3)		6 (54.5)		0		2 (25.0)		1 (20.0)	
	Other EU country	11 (18.0)	2 (9.5)		2 (13.3)		2 (18.2)		0		4 (50.0)		1 (20.0)	
	Non-EU country	33 (54.1)	13 (61.9)		11 (73.3)		3 (27.3)		1 (100)		2 (25.0)		3 (30.0)	
Living area *n* = 992 (95.4%) NA = 48 (4.6%)	A big city	310 (31.2)	85 (35.4)	14.6	70 (27.7)	18.6	56 (24.2)	21.8	37 (34.6)	26.8	48 (40.3)	13.8	14 (33.3)	10.1
	The suburbs or outskirts of a big city	189 (19.1)	30 (12.5)	14.6	54 (21.3)	12.6	33 (14.3)	7.8	5 (4.7)	3.7	55 (46.2)	26.0	12 (28.6)	20.5
	A town or a small city	258 (26.0)	63 (26.2)	40.7	74 (29.2)	31.7	61 (26.4)	25.6	40 (37.4)	30.8	6 (5.0)	31.2	14 (33.3)	48.6
	A country village	198 (20.0)	53 (22.1)	27.6	40 (15.8)	31.3	77 (33.3)	41.2	21 (19.6)	37.8	6 (5.0)	18.5	1 (2.4)	17.8
	A farm or home in the countryside	37 (3.7)	9 (3.8)	2.5	15 (5.9)	5.8	4 (1.7)	3.6	4 (3.7)	0.9	4 (3.4)	10.5	1 (2.4)	3.0
What is your living situation? *n* = 986 (94.8%) NA = 54 (5.2%)	Single or separated, living alone	246 (24.9)	72 (30.3)		49 (19.5)		59 (25.7)		10 (9.4)		44 (37.0)		12 (28.6)	
	Married or in a legally registered civil union, living with a partner	241 (24.4)	44 (18.5)		33 (13.1)		106 (46.1)		12 (11.3)		36 (30.3)		10 (23.8)	
	Living with a partner without being married or in a civil union	104 (10.5)	25 (10.5)		32 (12.7)		13 (5.7)		9 (8.5)		20 (16.8)		5 (11.9)	
	Having a partner, but not living with him/her in the same household	37 (3.8)	16 (6.7)		9 (3.6)		6 (2.6)		1 (0.9)		3 (2.5)		2 (4.8)	
	Living with parent(s)	321 (32.6)	72 (30.3)		121 (48.2)		37 (16.1)		66 (62.3)		13 (10.9)		12 (28.6)	
	Other	37 (3.8)	9 (3.8)		7 (2.8)		9 (3.9)		8 (7.5)		3 (2.5)		1 (2.4)	
People in household mean (sd) *n* = 980 (94.2%) NA = 60 (5.8%)		2.62 (1.48)	2.55 (1.58)	2.60 (1.32)	2.89 (1.44)	2.34 (1.32)	2.49 (1.51)	2.33 (1.30)	3.03 (1.35)	3.23 (1.53)	2.15 (1.19)	2.59 (1.35)	2.52 (1.57)	2.38 (1.31)
ESISCED *n* = 984 (94.6%) NA = 56 (5.4%)	1	36 (3.7)	5 (2.1)	2.2	11 (4.4)	19.8	16 (7.0)	9.7	3 (2.8)	3.0	0	9.2	1 (2.4)	24.0
	2	166 (16.9)	34 (14.3)	13.0	36 (14.3)	8.8	47 (20.6)	32.5	34 (31.8)	41.0	9 (7.6)	13.7	6 (14.6)	14.0
	3	167 (17.0)	61 (25.6)	40.9	30 (12.0)	24.5	48(21.1)	17.2	16 (15.0)	12.3	11 (9.2)	11.5	1 (2.4)	12.5
	4	178 (18.1)	31 (13.0)	3.9	67 (26.7)	17.8	17 (7.5)	7.3	28 (26.2)	19.2	31 (26.1)	23.0	4 (9.8)	12.8
	5	108 (11.0)	35 (14.7)	18.2	25 (10.0)	13.4	17 (7.5)	8.4	2 (1.9)	4.4	22 (18.5)	19.3	7 (17.1)	14.0
	6	122 (12.4)	22 (9.2)	7.8	27 (10.8)	4.4	27 (11.8)	9.7	9 (8.4)	5.0	25 (21.0)	11.5	12 (29.3)	10.1
	7	144 (14.6)	32 (13.4)	13.1	32 (12.7)	11.2	44 (19.3)	15.2	14 (13.1)	15.1	16 (13.4)	11.3	6 (14.6)	9.4
	Other	63 (6.4)	18 (7.6)	0.9	23 (9.2)	0.1	12 (5.3)	0.2	1 (0.9)	0.1	5 (4.2)	0.5	4 (9.8)	3.2
Working situation in the last seven days *n* = 937 (90.1%) NA = 103 (9.9%)	In paid work (or away temporarily)	496 (52.9)	132 (56.4)	49.1	103 (42.0)	47.0	125 (54.6)	48.9	35 (47.9)	48.3	74(63.8)	54.1	27 (67.5)	45.6
	In education (not paid for by employer), even if on vacation	151 (16.1)	52 (22.2)	10.0	45 (18.4)	6.2	27 (11.8)	5.3	8 (11.0)	10.1	14 (12.1)	12.0	5 (12.5)	5.0
	Unemployed and actively looking for a job	54 (5.8)	7 (3.0)	3.0	21 (8.6)	5.6	14 (6.1)	2.6	3 (4.1)	5.1	8 (6.9)	4.4	1 (2.5)	4.1
	Unemployed, wanting a job but not actively looking for a job	23 (2.5)	3 (1.3)	1.3	9 (3.7)	1.3	3 (1.3)	1.3	5(6.8)	1.8	2 (1.7)	0.8	1 (2.5)	1.6
	Permanently sick or disabled	55(5.9)	4(1.7)	2.4	9(3.7)	2.9	28 (12.2)	6.1	4 (5.5)	0.8	8(6.9)	3.5	2 (5.0)	4.1
	Retired	33 (3.5)	15 (6.4)	24.0	1 (0.4)	32.0	10 (4.4)	23.9	0	26.6	5 (4.3)	21.6	2 (5.0)	31.8
	In community or military service	2 (0.2)	0	0.0	0	0.0	0	0.0	2 (2.7)	0.0	0	0.1	0	0.0
	Doing housework, looking after children or other persons	14 (1.5)	3 (1.3)	9.3	5 (2.0)	4.1	4(1.7)	10.4	1 (1.4)	6.6	0 (0.0)	2.0	1 (2.5)	6.7
	Other	109 (11.6)	18 (7.7)	1.1	52(21.2)	0.9	18(7.9)	1.6	15 (20.5)	0.7	5(4.3)	1.6	1(2.5)	1.0
Working hours per week mean (sd) *n* = 750 (72.1%) NA = 290 (27.9%)		33.61 (12.74)	35.12 (11.61)	38.94 (15.02)	31.69 (12.73)	40.27 (13.40)	31.87 (12.89)	34.74 (14.47)	36.86 (16.17)	44.81 (12.62)	34.57 (12.93)	38.74 (12.46)	36.91 (9.59)	36.52 (15.42)
Feeling about household income *n* = 876 (84.2%) NA = 289 (15.8%)	Living comfortably on present income	339 (38.7)	96(42.3)	34.2	82(38.1)	28.4	85(40.3)	47.2	5(7.6)	8.0	55(47.4)	54.4	16(39.0)	37.5
	Coping with present income	393(44.9)	102(44.9)	51.5	83(38.6)	52.6	96(45.5)	39.8	51(77.3)	60.6	46(39.7)	33.2	15(36.6)	43.7
	Finding it difficult to live on present income	98(11.2)	24(10.6)	10.5	25(11.6)	17	22(10.4)	10.1	8(12.1)	27.9	11(9.5)	9.0	8(19.5)	13.7
	Finding it very difficult to live on present income	30(3.4)	5(2.2)	3.7	12(5.6)	1.9	7(3.3)	3.0	1(1.5)	3.5	3(2.6)	3.4	2(4.9)	5.0
	Other	16(1.8)	0(0.0)		13(6.0)		1(0.5)		1(1.5)		1(0.9)		0(0.0)	
Profession *n* = 948 (91.2%) NA = 92 (8.8%)	Currently not in paid employment	432(45.6)	88(36.7)		138(58.0)		100(46.3)		71(66.4)		29(27.9)		6(14.0)	
	Professional and technical (for example, doctor, teacher, engineer, artist, accountant)	160(16.9)	43(1 .9)		43 (18.1)		22 (10.2)		15 (14.0)		24 (23.1)		13 (30.2)	
	Higher administrator (for example, banker, executive in big business, high government official, union official)	32 (3.4)	14 (5.8)		2 (0.8)		8 (3.7)		2 (1.9)		4 (3.8)		2 (4.7)	
	Clerical (for example, secretary, clerk, office manager, bookkeeper)	78 (8.2)	19(7.9)		11(4.6)		19(8.8)		3(2.8)		19(18.3)		7(16.3)	
	Sales (for example, sales manager, shop owner, shop assistant, insurance agent)	30(3.2)	8(3.3)		8(3.4)		5(2.3)		3(2.8)		5(4.8)		1(2.3)	
	Services (for example, restaurant owner, police officer, barber, waitress, caretaker)	98(10.3)	33(13.8)		22(9.2)		25(11.6)		0		12(11.5)		6(14.0)	
	Skilled worker (for example, foreman, motor mechanic, printer, seamstress, electrician)	46(4.9)	18(7.5)		1(0.4)		23(10.6)		1(0.9)		1(1.0)		2(4.7)	
	Semi-skilled worker (for example, bricklayer, bus driver, cannery worker, carpenter, sheet metal worker, baker)	33(3.5)	10(4.2)		4(1.7)		5(2.3)		6(5.6)		6(5.8)		2(4.7)	
	Unskilled worker (for example, labourer, porter, unskilled factory worker)	33(3.5)	7(2.9)		7(2.9)		6(2.8)		6(5.6)		3(2.9)		4(9.3)	
	Farm worker (for example, farmer, farm labourer, tractor driver)	6(0.6)	0		2(0.8)		3(1.4)		0		1(1.0)		0	
Contact with support group in the last 12 months *n* = 974 (93.7%) NA = 66 (6.3%)	No	801(82.2)	193(80.8)		237(95.6)		145(64.7)		97(93.3)		99(84.6)		30(71.4)	
	Yes	173(17.8)	46(19.2)		11(4.4)		79(35.3)		7(6.7)		18(15.4)		12(28.6)	
Support through the internet *n* = 163 (94.2%) NA = 10 (5.8%)	No	61(37.4)	20(44.4)		4(57.1)		26(34.7)		1(14.3)		8(47.1)		2(16.7)	
	Once	14(8.6)	4(8.9)		0		9(12.0)		0		1(5.9)		0	
	A few times	49(3 .1)	11(24.4)		3(42.9)		24(32.0)		3(42.9)		5(29.4)		3(25.0)	
	Often	39(23.9)	10(22.2)		0(0.0)		16(21.3)		3(42.9)		3(17.6)		7(58.3)	
Support in face-to-face meetings *n* = 167 (96.5%) NA = 6 (3.5%)	No	27(16.2)	4(8.7)		2(28.6)		17(22.1)		1(14.3)		1(5.6)		2(16.7)	
	Once	40(24.0)	14(30.4)		0(0.0)		19(24.7)		1(14.3)		1(5.6)		5(41.7)	
	A few times	78(46.7)	16(34.8)		5(71.4)		35(45.5)		3(42.9)		14(77.8)		5(41.7)	
	Often	22(13.2)	12(26.1)		0		6(7.8)		2(28.6)		2(11.1)		0	

All participant characteristics are represented as total numbers (n) and percentages (%). Non-categorical parameters are shown as the mean and standard deviation (sd). NA: not available

The mean age of the participants was 32.4 years (SD 13.6), ranging from 25.2 (SD 10.1) in Poland to 39.4 (SD 14.7) in the Netherlands. The youngest participant was 16 years old, and the oldest was 75 years old. Compared with the general population (ESS) in their respective countries, the participants recruited in dsd-LIFE were 10–20 years “younger”, with the largest gap observed in Poland (mean age of the Polish population in ESS 46.1, SD 18.9).

In the country-specific analysis, the overall majority of participants were living as females (ranging from 59.8%, 73/122 in Sweden to 84.3%, 231/274 in France), except for in Poland (42.1%, 45/107), which was easily explained by varying recruitment numbers in the different diagnosis groups (Table 6). Regarding citizenship, the clear majority (more than 92%) hold citizenship in the country in which they live. This finding is very similar to the results from the ESS. Participants of dsd-LIFE were mainly living in large or small cities (ranging from approx. 45.3% in Sweden to 72.0% in Poland). Compared to ESS reference data, people living in large cities and suburbs were overrepresented in the dsd-LIFE population. Moreover, 6.4% of the participants had a migration background (first language spoken at home was not the same as the official language in the respective country).

*Living circumstances* varied among the countries (Table 6). The total “number of people living in the household” was similar across the study countries, with a mean between approximately two and three persons per household, and comparable to that of the general population (ESS).

In France and Poland, most of the participants were living with their parents (48.2% and 62.3%); in Sweden, the largest group of participants lived alone (37.0%); in Germany and the United Kingdom, the participants equally lived alone (30.3% and 28.6%), were married (18.5% and 23.8%) or lived with parents (30.3% and 28.6%), whereas in the Netherlands, most of the participants were married (46.1%).

Regarding *education* assessed via the ESS version of the International Standard Classification of Education^1^ (ESISCED; ranging from 1: basic education to 7: academic degree ≥ master), 20.5% of the DSD cohort from the dsd-LIFE cohort had a low education level (ESISCED ≤2), 46.1% had an intermediate education level (ESISCED 3–5), and 27.0% had high education levels, while the remaining 6.4% stated other education (e.g., school for children with learning difficulties). In general, the dsd-LIFE cohort had somewhat higher education levels than the ESS cohort with some differences across countries. In Germany, the distribution of categories was comparable to the ESS population, while in France, the Netherlands and more prominently in Sweden and the UK, we found a pattern of higher levels of education compared to the ESS cohort. Poland was the only example of somewhat lower educated participants compared to the general population.

Most subjects in the dsd-LIFE cohort performed paid *work* (52.9%) or had spent time in educational pursuits (16.1%) in the last 7 days. The remaining participants were either unemployed (8.3%), permanently sick or disabled (5.9%) or otherwise occupied in the last 7 days (retired, in community or military service, doing household work or other: 16.8%). This distribution was almost the same in all dsd-LIFE countries. Compared to the percentage reported in the ESS, the percentage of persons in paid work was very similar (e.g., Poland: 47.9% in dsd-LIFE vs. 48.3% in ESS), and the number of working hours per week was comparable among the dsd-LIFE countries (approx. 32–37 h, mean 33.6, SD 12.7). However, compared to those reported in the ESS, the number of working hours per week was 4 to 8 h less. In the small sample from the UK, there were more participants with full-time work and working hours comparable to the English ESS data, indicating some recruitment bias. The number of persons currently receiving education was two to three times higher in all countries, except in Poland and Sweden, than in the ESS (e.g., France: 18.4% in dsd-LIFE vs. 6.2% in ESS), which was explained by the lower mean age of the study population. In contrast, the proportion of retired people was much smaller in the dsd-LIFE cohort (e.g., France: 0.4% in dsd-LIFE vs. 32.0% in ESS).

Regarding *household income*, most of the dsd-LIFE participants were living comfortably or coping on their present income (both categories combined: 83.6%). This result was found for all dsd-LIFE countries (≥ 75% for combined categories). The results were also very similar to the country-specific outcomes of the ESS. The participants’ professions varied, but no ESS data for comparison were available. A detailed description is presented in Table 6.

In total, 17.8% of the dsd-LIFE cohort had contact with *support groups* in the last 12 months, varying between 4.4% in France and 35.3% in the Netherlands. Of these, 42–84% had contact via the Internet at least a few times, and 42–89% had contact in face-to-face meetings at least a few times.

#### Assessment of data quality

A total of 47 out of 1040 DSD participants (4.5%) did not respond to the patient-reported outcome questionnaires. The non-completion rate ranged from 0% (0/107) in Poland to 7.6% (19/231) in the Netherlands. A detailed overview of total non-response to the standardized instruments is shown in Table 7. The percentage of non-response to any entire questionnaire was 5–6% for the WHO-QOL-Bref, HADS, Adult ADHD Self-Report Scale (ASRS), Autism Spectrum Quotient (AQ10), RSES, and CSQ4. For the UGDS, the non-response rate for the entire questionnaire was 24% for the female version and 47% for the male version. Because several participants completed both female and male versions of this questionnaire, these percentages are based on all 1040 participants with DSD in the cohort. The Experiences in Close Relationships - Revised (ECR-RS) was not completed by 1.1% of subjects.^2^ The frequency of missing items and missing sub-scores in completed questionnaires was less than 1.5% except for the UGDS scales, where 24.1% (female version) and 38.5% (male version) of the items were missing from the completed questionnaires (data not shown). Medical history was mandatory and was supplied by 99.5% of the participants. Very few participants did not meet in person with the study nurse or the physician but were willing to answer the online PRO at home. These participants were included in the study because diagnoses and data on medical history could be obtained from the medical records.

**Table 7:** Frequencies of missing questionnaires, sub-scores and items of standardized instruments according to diagnosis groups

	% Non-response to entire questionnaire					
	Total	CAH	XY DSD	Klinefelter	Turner	Other^a^
WHO-QOL-Bref	4.9	4.9	2.7	5.5	5.7	6.9
HADS	5.1	4.9	2.7	6.0	6.0	6.9
ASRS	5.4	4.9	3.2	6.4	6.3	6.9
AQ10	5.4	4.9	3.2	6.4	6.3	6.9
Rosenberg self esteem	5.6	4.9	3.6	6.4	6.6	6.9
CSQ4	6.3	4.9	3.2	8.3	7.0	11.0
UGDS female	24.3	14.2	16.2	44.0	22.3	30.1
UGDS male	47.2	51.1	33.9	32.1	65.8	43.8
ECR-RS^b^	1.1	0.0	2.5	1.8	0.5	0.0

^a^These include the groups 47,XYY, XX gonadal dysgenesis, XX ovotestis, 46,XX, and 45,X/46,XY + other Y

^b^The ECR-RS was only answered by participants who answered “yes” to the question: “Have you ever had a “romantic” relationship?” A total of 700 participants (67.3%) answered yes, while 273 (26.3%) answered “no”, and 67 (6.4%) did not answer the question

A total of 928 (89.2%) of the 1040 DSD participants underwent at least part of the physical examination. Participation in the general examination by diagnosis group, country and gender is shown in Table 8. No relevant differences in the subgroups were found except for a lower participation rate in Sweden (65.6%, 80/122), whereas in Poland and the United Kingdom, all participants had at least a partial general examination. Participation in the gynaecological and urological examination is also shown in Table 8. Approximately one-half (346/717, 48.3%) of the females and 71.1% (221/311) of the males took part in the respective examinations. Across the countries, there was large variation in participation in gynaecological and urological examinations, reaching from none in the UK to almost all participants in Poland (both males and females). Participation in the examinations across diagnosis groups was more evenly distributed around the respective mean participation rate with the exception of the more frequent gynaecological examination in XY DSD females (92/142, 64.8%). In the group of XX CAH males, only 1 out of 5 took part in the urological examination. Metabolic parameters for 886 persons (85.2%) and hormones for 772 participants (74.2%) were determined. Ultrasound was performed on 380 (36.5%) of all 1040 DSD participants,^3^ a spermiogramme was evaluated for 50 participants, a Dexa scan was carried out on 613 (58.9%) participants, and a BIA was performed on 283 participants (27.2%). Carotid IMT measurements were taken on 208 participants (20.0%).

**Table 8:** Participation in medical exams: general physical examination, gynaecological exam, urological exam per diagnosis group, country and gender

	General physical examination	Gynaecological examination^a^	Urological examination^c^
	Participation: total sample (%)	Participation: female gender (%)	Participation: male gender (%)
Diagnosis group			
Turner syndrome	276/301 (91.7%)	123/301 (40.9%)	Not applicable
Klinefelter syndrome	186/218 (85.3%)	0/1	144/212 (32.1%)
XY DSD conditions	191/222 (86.0%)	92/142 (64.8%)	59/73 (80.8%)
CAH	210/226 (92.9%)	103/221 (46.6%)	1/5 (20.0%)
Other conditions^a^	65/73 (89.0%)	28/52 (53.8%)	17/21 (81.0%)
Country			
Germany	236/244 (96.7%)	94/182 (51.6%)	46/56 (82.1%)
France	258/274 (94.2%)	139/231 (60.2%)	32/43 (74.4%)
The Netherlands	204/250 (81.6%)	23/153 (15.0%)	59/97 (64.8%)
Poland	107/107 (100%)	44/45 (97.8%)	61/62 (98.4%)
Sweden	80/122 (65.6%)	46/73 (63.0%)	23/49 (46.9%)
The United Kingdom	43/43 (100%)	0/33	0/10
Gender			
Female	647/717 (90.2%)	346/717 (48.3%)	^c^
Male	270/311 (86.8%)	^b^	221/311 (71.1%)
Other	11/12 (91.7%)	^b^	^c^

^a^These include the groups 47,XYY, XX gonadal dysgenesis, XX ovotestis, 46,XX males, and 45,X/46,XY + other Y

^b^Participation in gynaecological examination was evaluated only in females. Nevertheless, one male participant in Germany and in Poland as well as three participants with other gender received a gynaecological examination

^c^Participation in urological examination was evaluated only in males. Nevertheless, one female participant in Poland as well as four participants with other gender received a urological examination

Retrospective data regarding the specific condition and treatment were available for 480/1040 (46.2%) of the participants. For the Turner group (152/301, 50.5%), the XY DSD group 108/222, 48.6%), the CAH group (131/226, 57.9%) and the smaller groups (including 47,XYY, XX DSD, 46, XX, and 45,X/46,XY + other Y: 46/73, 63.0%), the availability of at least some retrospective data was approximately the same. For the Klinefelter group, the availability of data was considerably lower (43/218, 19.7%). Gonadal histology was available for 123/1040 participants (11.8%), and for 500/1040 subjects (48.1%), self-reported information or data from medical reports were available regarding former condition-specific surgeries.

## Discussion

### The study sample

The dsd-LIFE consortium was able to recruit a large comprehensive DSD study population with 1040 participants. The initial sample size calculation had anticipated 1500 participants, but the size of the subgroups of the 1040 participants are large enough for adequate statistical analysis. It was easiest to recruit female participants with Turner syndrome and congenital adrenal hyperplasia (CAH) and men with Klinefelter syndrome and sample size was sufficient in these groups. The number of recruited participants with these specific diagnoses is consistent with the incidence of the diagnoses. Most patients were recruited with Turner syndrome (1:2500) followed by Klinefelter (1:500–1000), CAH (1:15,000) and XY DSD conditions (1:25–150,000). The group of XY DSD cases included the least common diagnoses that were clinically and/or genetically sub-classified. A total of 222 participated with only one third living in male gender (*n* = 73), covering a wide range of clinical diagnoses. Despite prolongation of the recruitment period for this group, it was most difficult to engage participants with XY-DSD conditions except for the centres in France, Poland and the Netherlands, which had specialized clinics for this group. Sufficient numbers for statistical analysis were achieved for the diagnoses of complete androgen insensitivity (CAIS), partial androgen insensitivity (PAIS), XY gonadal dysgenesis and 45,X/46,XY conditions but not for XY androgen synthesis defects. We conclude that despite the labelling of the study as dsd-LIFE, the group most closely associated with the term “disorders of sex development” was the most difficult to recruit. Adults with the condition appear to have less access to specialized centres or clinics, and appropriate treatment appears to be least efficient in this group [46]. We speculate that less regular contact and more variation in health care among this group may also contribute to some alienation. Equally, we were unable to recruit a sufficient number of participants with XX GD. We used mixed ways of recruitment via the medical centers, website, support groups with the aim to reach as many as possible individuals with rare diagnoses. Moreover, analysis of contact/membership of a support group as an influencing factor on outcomes is planned. It could be shown that the most efficient means of recruitment were direct contact and information provision about the study in the clinics, leading to a participation rate of 76%. The least successful means of recruitment was contact by mail, through support groups and website, with a participation rate of 22%, 24% and 0% respectively.

All participants could be classified according to the Chicago classification. However, for some participants with very unusual clinical presentation or unusual chromosomal or genetic findings, correct classification became a challenge. This was relevant, for example, in the 45,X/46,XY subgroup presenting with a non-androgenized Turner phenotype and individuals with androgenization. The latter group clinically resembles the XY DSD conditions and could be assigned to this group for most analyses. Similarly, the classification of 46,XX males and 47,XYY males proved to be difficult: clinically, these individuals are similar to Klinefelter males because they all display testicular dysgenesis and could be included in this group for outcome analyses. Moreover, the 46,XX gonadal dysgenesis patients included in the XX DSD subgroup are very different from 46,XX females with androgen effects due to CAH and should be considered as a separate group. Finally, 46,XX individuals with 21-OHD or 11ß-OHD deficiency living as males may be included or excluded in outcome analyses depending on the specific research question. We concede that the classification system from the Chicago Consensus Conference was very helpful in terms of a conceptual frame of reference. However, individual cases may still be difficult to allocate to one of the major groups. Any classification must be adapted to the purpose at hand, be it research, patient care or public communication.

### Participation in different parts of the study

#### Participation in medical history and retrospective chart review

Partial to complete data on contemporary and past medical history from personal encounters with the physicians were obtained for 99.5% of the participants. Retrospective medical data from the chart review addressing the time of diagnosis and past treatment were obtained for only 46.2% of the participants. In the centres focusing on adult care, charts from previous treatment in childhood were not available. Moreover, for older participants, previous charts were not available because they were destroyed after 30 years in accordance with hospital regulations.

#### Participation in medical examinations

Most of the dsd-LIFE participants did take part in the general examination, with no relevant differences among diagnosis groups, gender or country. The participation rate in gynaecological or urological examinations varied between 15 and 97.8% and 46.9–98.4%, respectively. The participation rate in these intimate exams was highest in the XY DSD group, which may be interpreted as an indication of these participants’ need for gynaecological/urological follow-up and counselling. The participation rate in the Dexa scans (58.9%) was high, demonstrating that bone health is an issue for many persons with DSD conditions. The participation rate was lowest for the additional exams, such as genital ultrasound (36.5%), BIA (27.2%), carotid IMT (20%) and spermiograms (4.8%).

#### Response rate of the PRO

The rate of non-response to the entire PRO part of the dsd-LIFE was very low (4.5%). Especially, the standardized instruments used in the study showed almost complete response rates. The only exception was the UGDS, which had a non-response rate of 24.3% for the female and 47.2% for the male version. It is not entirely clear why questions regarding gender dysphoria were so often left unanswered. The UGDS was originally designed for transgender individuals without DSD but has been used without problems for people with DSD as well [47]. The low rate of responses to this particular instrument might indicate that there were misunderstandings or confusion regarding the items among our participants. Originally, the scale was presented in two gender-specific versions for those who live as female gender and those who live as male gender. In contrast to this presentation, i.e., in the German network study, the dsd-LIFE consortium wanted to allow people with non-binary identities (not identifying as either male or female) [48] to respond to items of both the male and female versions. This, however, may have resulted in people considering many questions as not applicable. For instance, persons identifying as females—most of our sample—may have omitted answers to all questions for males, but, being in a non-answering mode (the first half of the questionnaire considered questions for males), they may have omitted answers to questions for females as well.

We conclude that in general, most participants showed a very positive attitude toward answering the questionnaires and sharing their views on QoL, psychological well-being, treatment experiences, sexuality and ethical considerations.

#### Homogeneity/heterogeneity of countries

The characteristics that were heterogeneous among the dsd-LIFE countries were age, gender and diagnosis. Unequal distributions of diagnoses, age groups and gender per country may be explained by varied focuses of clinical care, research interests or access of dsd-LIFE study personnel to clinics in the centres. Therefore, the condition groups were not evenly distributed among all centres; all analysis concerning country differences must be controlled for the effects of the diagnosis, and vice versa.

#### Representativeness and limitations

The study sample is a convenience sample recruited by the specialized study centres with different clinical foci and in a variable proportion per country via support groups. A participation rate of 36% of all eligible persons in each centre cautions against generalizability. The participation rate ranged from 30.0% in the Netherlands to 54.4% in Sweden. Reasons for these differences are most likely explained by different approaches towards recruitment. Compared to the ESS sample, the sample for the current study was younger; the mean age was 32.36 years (SD 13.57) compared to the mean age of the ESS, which ranged from 48.70 years (SD 18.57) in Germany to 51.83 years (SD 19.12) in the United Kingdom. These age differences might be explained by the fact, that only in the recent years specialized clinics for adults with DSD conditions have been installed in several European countries. Patients are refered there mainly through transition programmes. Subsequently, few older patients are followed in these clinics. Most participants in the current analysis lived in an urban environment (large city to town), had a slightly higher education level and were satisfied or coping well on their present income. The sample size of females was approximately double that of males. Only a few participants reported to have a gender other than male or female; those participants were recruited in Germany and the Netherlands. In those two countries, there is—perhaps more than in the other countries—discussion in support groups and among the general public on the acknowledgement of non-binary identities. For instance, in a Dutch study on the prevalence of gender dysphoria, 3–4% reported to have an ambivalent gender identity (equal identification with the male and female genders) [49]. However, a selection bias such as that in our study is well known from previous studies in clinical samples. This study has attempted to document at least the number of potential participants from hospital data identifying potential participants, who were all approached and informed. Through this process, numbers of contacted persons, ways of contact and participation rate could be retrieved. Unfortunately, specific information on the diagnoses of non-participants and their reasons not to participate could not be obtained because the ethical restrictions did not allow any data on non-participants to be collected. We are therefore unable to provide a non-responder analysis.

### Data quality

Overall, the data quality of the study is good because the mandatory parts of the study, such as medical history and PRO, showed very low rates of completely missing data of 0.5% and 4.5%, respectively. Subsequently, for most analyses of the cohort, there were ample data for statistical analysis. In addition, for most rare diagnoses, such as 45,X/46,XY, CAIS, PAIS, partial XY gonadal dysgenesis and CAH, data are estimated to be adequate for clinical recommendations. Only for some very rare diagnoses, such as XY complete gonadal dysgenesis, XX gonadal dysgenesis and androgen synthesis defects, were the numbers of participants and data insufficient for statistical analysis. However, these rare diagnoses are of special interest and will be described as case series.

### Analyses and publication strategy

The main data analyses for the primary objectives will be performed by the coordination centre for clinical studies of the Charité Universitätsmedizin Berlin. Secondary analyses addressing specific areas of interest or related to clinical subgroups will be performed by author teams composed of members of the dsd-LIFE consortium based on a statistical analysis plan and a written publication proposal. In addition, the results will be presented to the dsd-LIFE recommendation groups that augment the existing knowledge on health care for these conditions with new information from the study. Dissemination strategies will include scientific publications, presentations within the scientific community and associations of health care professionals, support groups, and other stakeholders and policymakers.

## Conclusions

dsd-LIFE is the first and largest European cross-sectional study including the majority of conditions encompassed by the DSD classification. The data analyses will focus on issues that are important for the improvement of care and the development of clinical recommendations for patients with these conditions, such as hormone therapy, surgery, fertility, psychological and social support, and psychosexual and ethical issues. Moreover, the specific aspects of single conditions, such as Turner syndrome and Klinefelter syndrome, 45,X/46,XY, CAIS, PAIS and CAH will be considered for improvement of care. A special focus will be to analyse and consider the participants’ views on the different issues. Altogether, QoL, psychosexual issues, physical and mental health and satisfaction with treatment, support and helath services will be measured in the whole cohort and in sub-samples if indicated. The data from this large sample will provide a sufficient basis for evidence-based recommendations for improvement of clinical care of individuals affected by a DSD condition.

## Abbreviations

11ß-OHD: 11ß-hydroxylase deficiency
21-OHD: 21-OH deficiency: 21-hydroxylase deficiency
46,XX DSD: 46,XX disorders/differences of sex development
46,XY DSD: 46,XY disorders/differences of sex development
AMH: Anti-muellerian hormone
AQ10: Autism Spectrum Quotient (AQ)
ASRS: Adult ADHD Self-Report Scale
BIA: Bioelectrical impedance analysis
CAH: Congenital adrenal hyperplasia
CAIS: Complete androgen insensitivity
CHC-SUN: Child Health Care – Satisfaction, Utilization, Needs Questionnaire
CRF: Case report form
CSQ4: Client Satisfaction Questionnaire
DEXA: Dual-energy X-ray absorptiometry
DSD: Disorders/differences of sex development
ECR-RS: Experiences in Close Relationships - Revised
ESISCED: European Social Survey version of the International Standard Classification of Education
ESS: European Social Survey
HADS: Hospital anxiety and depression scale
HRQOL: Health-related quality of life
I-DSD: International DSD database
IMT: Intima media thickness
KKS: Koordinierungszentrum für klinische Studien
PAIS: Partial androgen insensitivity
PRO: Patient-reported outcome
QoL: Quality of life
R: Software environment for statistical computing
SAS: Statistical analysis software
SOP: Standard operational procedures
STC: Steering committee
UGDS: Utrecht Gender Dysphoria Scale
WHO-QOL-Bref: World Health Organization Quality of Life-Bref is a shorter version of the original instrument to assess Quality of Life

## Footnotes

1 ESISCED categories: 1: less than lower secondary; 2: lower secondary; 3: lower tier upper secondary; 4: upper tier upper secondary; 5: advanced vocational, sub-degree; 6: lower tertiary education, BA level; 7: higher tertiary education, ≥ MA level

2 The ECR-RS could be answered only by participants who answered ―yes‖ to the question: ―Have you ever had a “romantic” relationship?‖ A total of 700 participants (67.3%) answered yes, while 273 (26.3%) answered ―no‖ and 67 (6.4%) did not answer the question.

3 In France, one participant received both ultrasound examinations.
